# Internal Carotid Artery Dissection with Lidocaine Nerve Block Injection Trauma: A Rare Case Report

**DOI:** 10.7759/cureus.2027

**Published:** 2018-01-05

**Authors:** Naureen Narula, Faraz Siddiqui, Nakul Katyal, Akshay Avula, Michel Chalhoub

**Affiliations:** 1 Internal Medicine, Staten Island University Hospital; 2 Pulmonary and Critical Care, Staten Island University Hospital; 3 Neurology, University of Missouri Columbia

**Keywords:** internal carotid artery dissection, dental trauma, periodontal infection, injection trauma

## Abstract

Internal carotid artery dissection (ICAD) accounts for 25% of cerebrovascular accidents in young and middle-aged patients. Dissection occurs when the intimal wall of an artery is damaged as a result of trauma or defect. ICAD development after dental work is a relatively uncommon phenomenon. Our study highlights a rare presentation of ICAD that resulted from a direct lidocaine nerve block injection in a patient undergoing pulpotomy for a right maxillary second premolar tooth. We have described the case and reviewed the literature on this rare but potentially life-threatening phenomenon.

## Introduction

Internal carotid artery dissection (ICAD) represents an important cause of cerebrovascular accidents in young and middle-aged patients [1]. About 25% of strokes in patients under the age of 45 are related to cervicocephalic artery dissections [2-3]. The median time to appearance of symptoms is four days; thus, the diagnosis can be delayed [2, 4]. The disease was first recognized by Dratz in 1947 when he reported a case of ICAD as a complication from a direct injection to the carotid artery [4]. Cervical arterial dissection occurs when the intimal wall of an artery is damaged as a result of trauma or defect. As blood fills the layers of the arterial wall, thrombi form, which can lead to stroke, pseudoaneurysm, vessel occlusion, and stroke.

In this study, we present a case of ICA dissection that resulted from a direct lidocaine nerve block injection in a patient undergoing pulpotomy for a right maxillary second premolar tooth. We describe the case and review the literature on this rare but potentially life-threatening phenomenon.

## Case presentation

**Figure 1:**
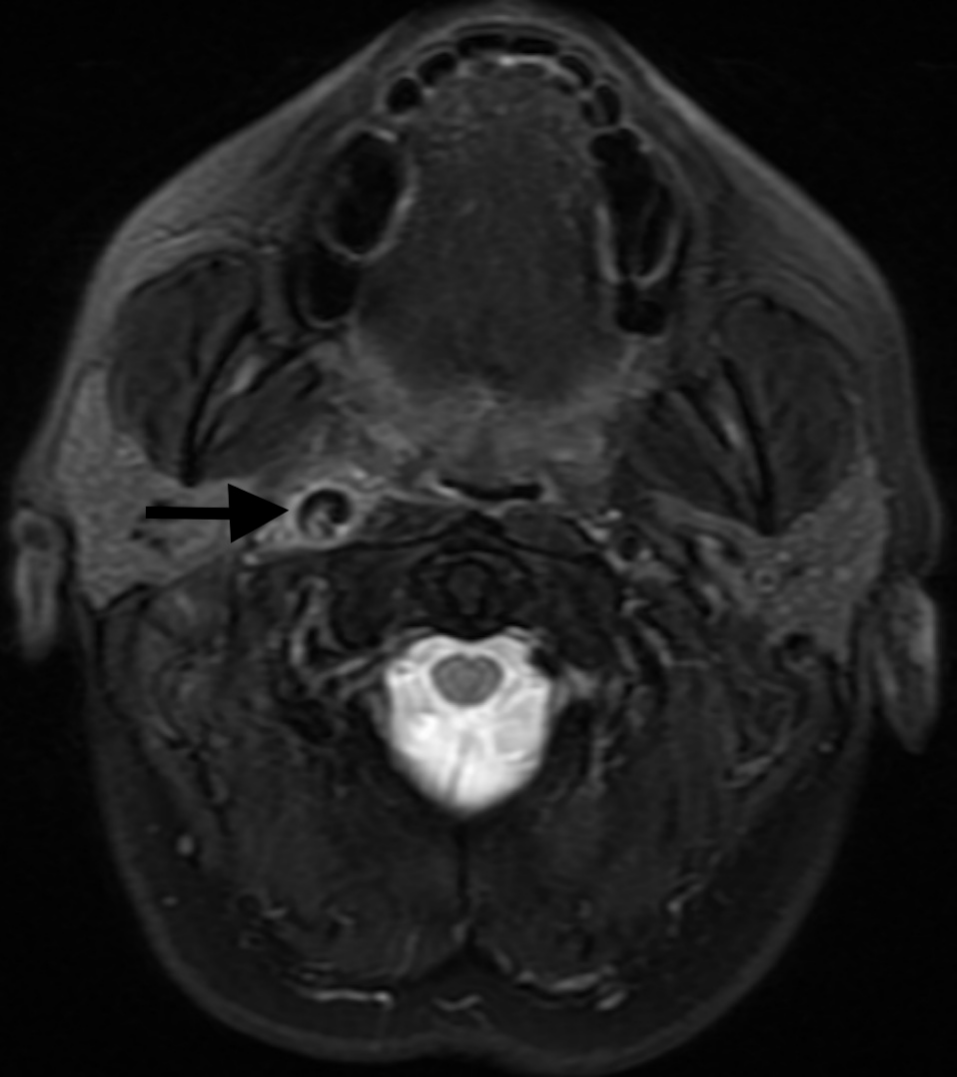
Magnetic Resonance Imaging (MRI) Brain MRI brain showing right distal internal carotid artery dissection. A notable dissection flap, along with mild inflammatory type signal changes, surrounding the carotid artery is seen (arrow).

A 58-year-old female presented to the emergency department (ED) with severe occipital headache associated with right-sided neck pain and increased sleepiness of five days' duration. Her headache was reportedly 10/10 in intensity and was associated with nausea, drowsiness, and dizziness. She denied having a change in vision, hearing, or history of head or neck trauma. Her past medical history was significant for hypertension, atrial fibrillation, and dyslipidemia. Her medications consisted of Xarelto and she was treated with ablation in the past. Vital signs in ED showed a blood pressure (BP) of 206/98 mmHg, a temperature of 98°F, heart rate of 59 bpm, and a respiratory rate of 14/min. The examination was pertinent for right eye ptosis and right pupil constriction. She recalled that her symptoms started about a week ago when she received lidocaine injection prior to pulpotomy for her upper #5 tooth. Immediately after the procedure, she experienced a severe headache, profuse sweating, and shortness of breath. The dentist, therefore, was unable to complete the procedure and she was asked to follow-up after the symptoms resolved. After few days, she developed right-sided facial swelling and presented back to the ED where she underwent incision and drainage for a right-sided dental abscess. Antibiotic therapy was subsequently started. Two days later, she returned to the ED with above-mentioned chief complaints. Computed tomography (CT) head showed no acute changes. Magnetic resonance imaging (MRI) brain showed right distal ICA dissection with mild inflammatory type signal changes surrounding the carotid artery (Figure 1).

**Figure 2:**
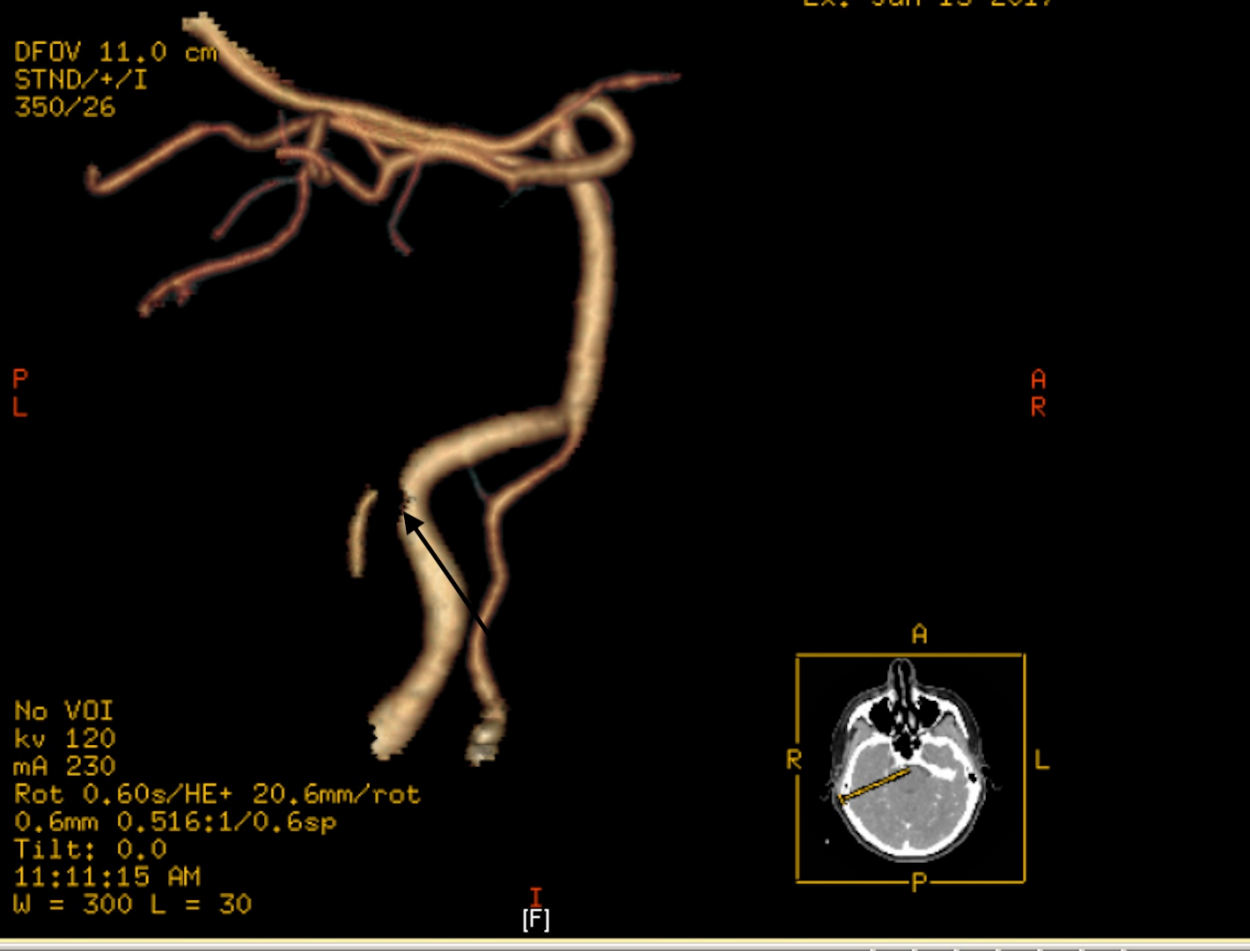
Computed Tomography Angiography (CTA) Neck CTA neck showing dissection of the right internal carotid artery at the C1-C2 level associated with infiltrative changes within the right carotid space

Computed tomography angiography (CTA) scan of the neck confirmed a dissection of the right internal carotid artery (ICA) at the C1-C2 level associated with infiltrative changes within the right carotid space (Figure 2).

She was started on intravenous (IV) heparin. A blood pressure reduction goal of 25% per day was decided. IV heparin was continued for three days and later the anticoagulation was switched to Xarelto. During the course of hospitalization, her symptoms gradually improved. She was discharged home on Xarelto after 12 days of hospital stay and was encouraged to follow-up with vascular surgery, cardiology, and neurology on an outpatient basis.

## Discussion

In this report, we have highlighted a rare presentation of ICAD that developed following a direct lidocaine injection trauma. We consider the possibility of a direct intraprocedural lidocaine injection needle-related internal carotid artery injury, as described by De Santis, et al. in a similar presentation [5]. The classical presentation of ICAD includes a triad of pain on one side of the head, face, or neck accompanied by a partial Horner's syndrome [6]. However, this classic triad is found in less than one-third of the patients; thus, in presence of any two elements of this triad, a diagnosis should be strongly suspected. Pain is usually the initial manifestation of ICAD [7]. The median time to appearance of other symptoms was usually around four days. Oculosympathetic palsy, consisting of miosis and ptosis, has long been recognized as a classical manifestation of ICAD but it is seen in less than half of the cases [8]. In our patient, symptoms developed after the anaesthetic block injection. There was no history of significant trauma, genetic predisposition, or other precipitating events; therefore, one can attribute that the ICAD developed secondary to the lidocaine injection trauma. Although the carotid neurovascular bundle anatomically is relatively distant from the anesthetic block target areas, there is always a possibility of an erroneous needle insertion more posteriorly and medially in the proximity of the neurovascular carotid bundle, causing an internal carotid artery injury [5]. Also, cervical hyperextension and fully opened mouth during the dental procedure can contribute to the development of ICAD as this position can compress the cervical internal carotid artery against upper cervical vertebra and facilitate the needle reaching the submandibular capsule. Furthermore, in cases of needle insertion behind the second molar level, it may reach the posterior border of the mylohyoid muscle and penetrate into the carotid triangle. Periodontal infection also contributes majorly in pathogenesis [9-10]. Infection is associated with secretion of inflammatory cytokines, free radicals, and proteases that can lead to vessel wall weakening and increased susceptibility for rupture [9-10]. Given all these circumstances, the possible role of direct injection trauma in ICAD development cannot be excluded. To prevent thromboembolic complications, anticoagulation with intravenous heparin, followed by oral warfarin, has been recommended for all patients with acute dissections of the carotid or vertebral artery, regardless of the type of symptoms, unless there are contraindications, such as an intracranial extension of the dissection [6]. Spontaneous ICA dissections usually have a favorable prognosis, with approximately 90% of cases resolving without serious sequelae [7].

## Conclusions

Our case highlights a rare presentation of ICAD resulting from a lidocaine nerve block injection trauma and now adds to the limited number of cases in the literature describing this uncommon phenomenon. ICAD development after dental work is a rare but potentially life-threatening clinical scenario; therefore, precaution should be taken in managing patients with periodontal infections to minimize the risk of arterial injury.

## References

[1] Bang OY (2014). Intracranial atherosclerosis: current understanding and perspectives. J Stroke.

[2] Bogousslavsky J, Pierre P (1992). Ischemic stroke in patients under 45. Neurol Clin.

[3] Lee VH, Brown RD Jr, Mandrekar JN, Mokri B (2006). Incidence and outcome of cervical artery dissection: a population-based study. Neurology.

[4] Dratz HM, Woodhall B (1947). Traumatic dissecting aneurysm of left internal carotid, anterior cerebral and middle cerebral arteries. J Neuropathol Exp Neurol.

[5] De Santis F, Martini G, Thüringen P (2012). Internal carotid artery dissection after inferior alveolar nerve block for third molar dental care presented as hypoglossal nerve palsy. Vasc Endovascular Surg.

[6] Schievink WI (2001). Spontaneous dissection of the carotid and vertebral arteries. N Engl J Med.

[7] Silbert PL, Mokri B, Schievink WI (1995). Headache and neck pain in spontaneous internal carotid and vertebral artery dissections. Neurology.

[8] Venketasubramanian N, Singh J, Hui F, Lim MK (1998). Carotid artery dissection presenting as a painless Horner’s syndrome in a pilot: fit to fly?. Aviat Space Environ Med.

[9] Grau AJ, Brandt T, Buggle F (1999). Association of cervical artery dissection with recent infection. Arch Neurol.

[10] Guillon B, Berthet K, Benslamia L (2003). Infection and the risk of spontaneous cervical artery dissection. A case-control study. Stroke.

